# Economics in “Global Health 2035”: a sensitivity analysis of the value of a life year estimates

**DOI:** 10.7189/jogh.07.010401

**Published:** 2017-06

**Authors:** Angela Y Chang, Lisa A Robinson, James K Hammitt, Stephen C Resch

**Affiliations:** 1 Department of Global Health and Population, Harvard T.H. Chan School of Public Health, Boston, MA, USA; 2 Center for Health Decision Science, Harvard T.H. Chan School of Public Health, Boston, MA, USA; 3 Center for Risk Analysis, Harvard T.H. Chan School of Public Health, Boston, MA, USA; 4 Toulouse School of Economics, University of Toulouse Capitole, Toulouse, France

## Abstract

**Background:**

In “Global health 2035: a world converging within a generation,” *The Lancet* Commission on Investing in Health (CIH) adds the value of increased life expectancy to the value of growth in gross domestic product (GDP) when assessing national well–being. To value changes in life expectancy, the CIH relies on several strong assumptions to bridge gaps in the empirical research. It finds that the value of a life year (VLY) averages 2.3 times GDP per capita for low– and middle–income countries (LMICs) assuming the changes in life expectancy they experienced from 2000 to 2011 are permanent.

**Methods:**

The CIH VLY estimate is based on a specific shift in population life expectancy and includes a 50 percent reduction for children ages 0 through 4. We investigate the sensitivity of this estimate to the underlying assumptions, including the effects of income, age, and life expectancy, and the sequencing of the calculations.

**Findings:**

We find that reasonable alternative assumptions regarding the effects of income, age, and life expectancy may reduce the VLY estimates to 0.2 to 2.1 times GDP per capita for LMICs. Removing the reduction for young children increases the VLY, while reversing the sequencing of the calculations reduces the VLY.

**Conclusion:**

Because the VLY is sensitive to the underlying assumptions, analysts interested in applying this approach elsewhere must tailor the estimates to the impacts of the intervention and the characteristics of the affected population. Analysts should test the sensitivity of their conclusions to reasonable alternative assumptions. More work is needed to investigate options for improving the approach.

In its 2013 report, “Global health 2035: a world converging within a generation,” *The Lancet* Commission on Investing in Health (CIH) recommended the use of a “full income” approach to incorporate the benefits of health improvements into national accounts [1]. Under this approach, the value of changes in life expectancy are added to the value of changes in gross domestic product (GDP) to capture the effects of health improvements at the population level.

To support this approach, prominent members of the Commission, including economists Dean Jamison, Lawrence Summers, and Kenneth Arrow, developed an innovative method for estimating the value of an increase in population life expectancy and translating the results into an average value of a life year (VLY) [1]. They adjust a US estimate of the value of mortality risk reduction, assuming this value is proportional to GDP per capita and to remaining life expectancy. In their main result, they decrease the value for children aged 0 through 4 by 50 percent, then divide the total by the life expectancy gain to estimate VLY. This VLY is 2.3 times 2000 GDP per capita for life expectancy gains experienced by low– and middle–income countries (LMICs) from 2000 to 2011, assuming the gain is permanent, or 3.0 times GDP per capita without the reduction for young children. The CIH also reports the value of life expectancy gains for other country groups and time periods using the same approach, and applies the results to estimate the benefits of the interventions it recommends.

This VLY is within the range of the illustrative estimates of 1.0 to 3.0 times GDP per capita referenced by the 2001 Commission on Macroeconomics in Health (CMH), currently used as cost–effectiveness thresholds in the World Health Organization’s Choosing Interventions that are Cost–Effective program and elsewhere [2,3]. The CMH multipliers were intended as rough estimates of the value an average individual might place on an incremental change in life expectancy [4]. In contrast, the CIH multiplier is based on a specific historical gain.

The CIH faced many challenges in developing its approach. We examine the sensitivity of the 2.3 times GDP per capita estimate highlighted in its main report to changes in the parameter values [1]. Our goal is to investigate the effects of plausible alternative assumptions and to provide insights into the uncertainties in the results as well as the issues that arise in tailoring the approach for application elsewhere.

## General framework

Conceptually, the CIH approach is intended to reflect the value that members of a population place on specific changes in their own life expectancies. In this context, money is not of interest per se: it measures the extent to which individuals are willing to trade consumption of other goods and services for a life expectancy increase. Because these preferences are likely to vary across individuals and societies, ideally such analyses would rely on estimates from the affected population. However, due to gaps and inconsistencies in the empirical research, these values must be extrapolated from studies of other populations that differ in significant respects.

The CIH starts with an estimate of the value per statistical life (VSL), which represents a population average of individuals’ marginal rates of substitution between money and mortality risk in a defined time period [5]. Conventionally, VSL is estimated by dividing empirical estimates of individual willingness to pay for a small change in one’s own risk by the risk change.

The associated value per statistical life year (VSLY) could be estimated directly by researching the values individuals place on an increase in life expectancy, but few empirical studies exist. Instead, VSLY is usually estimated as a constant, by dividing average VSL by the remaining (discounted) life expectancy for the average individual [6,7]. Valuing current mortality risk as the product of a constant VSLY and remaining life expectancy yields values that decrease with age.

The CIH also derives a value per life year from a VSL estimate, but uses a different approach, as described in detail in the Methods section. The result is the average value of a specified population increase in life expectancy. Analysts who are interested in applying the CIH approach would need to follow the same steps to calculate the VLY associated with a particular policy. We follow the CIH practice and use the term “VLY” to distinguish their population–based quantity from the conventional VSLY.

Monetary values for improved survival are expected to vary with characteristics of the affected population and the mortality risks. The effects of many characteristics are not well–understood; there are significant inconsistencies and gaps in the available research even for high–income countries [8,9]. In its analysis, the CIH focuses on the variation associated with income and age–specific life expectancy.

Most VSL studies address high–income countries [10–12]. Research on the relationship between income and VSL generally confirms that VSL increases with income, as anticipated. However, the proportional change in VSL in response to a proportional change in income (ie, its income elasticity) is uncertain. While theory and empirical studies generally support elasticities greater than one, some studies report elasticities as high as two or three while others report values less than one [13]. Income elasticity is of particular importance when extrapolating from very high to very low income countries because the range of potential elasticities may affect the resulting VSL by orders of magnitude. Theoretical models suggest VSL will exceed the present value of future income or consumption because VSL reflects non–monetary benefits of living in addition to the monetary value of consumption and future earnings. Thus the income–adjusted VSL should not fall below the present value of income or consumption [13].

The theoretical and empirical evidence on the relationship between VSL and age or life expectancy is inconsistent, and generally does not support the hypothesis that VSL is proportional to (discounted) life expectancy nor the application of a constant VSLY [14,15]. Some studies find an inverse–U relationship between VSL and age for working–age adults, consistent with the prediction of life–cycle models [16–18]. Other studies, which include adults older than working age, find that values may decrease or remain constant with age [19,20].

Much of the research in higher–income countries suggests that adults value reducing risks to children more than to themselves [21–23]. The CIH posits, however, that particularly for countries with relatively low life expectancies, where much of the life expectancy gain results from decreased infant and child mortality, “[i]t is plausible that many societies and individuals will value reducing death rates at very young ages less than reducing death rates among, say, 25 year olds.” [1]. The CIH notes that empirical evidence supporting such lower values is “limited.”

## METHODS

The CIH faced two major challenges. First, most empirical estimates of the value of mortality risk reductions focus on high–income countries, while the CIH was interested in values for LMICs. Second, the available values are for a reduction in current mortality risk, while the CIH was interested in the value of life extension.

The CIH approach to the first challenge is relatively straightforward. They begin by dividing a VSL estimate by 10 000 to yield the value of a standardized mortality unit (VSMU) in 2011 dollars. They use “SMU” to refer to a 1 in 10 000 risk reduction for the current year and “VSMU” to refer to individual willingness to pay for this risk change. They use this terminology in part because the VSL concept is widely misunderstood [24]. It is not the value of saving a life with certainty; rather the VSL is derived from the value that an individual places on small changes in his or her own mortality risk, such as a 1–in–10 000 reduction.

The CIH states that it is reasonable to assume that the US VSMU was 1.8 percent of US GDP per capita in the year 2000, and that this ratio is constant across time and populations. In other words, they assume an income elasticity of 1.0: a one percent change in GDP per capita is associated with a one percent change in VSL and VSMU. For LMICs, the CIH uses the average of 2000 and 2011 GDP per capita, estimated as US$ 2718 and US$ 4201, respectively.

The CIH approach to the second challenge is more complex, and differs from the approaches commonly used. To estimate the value of a change in population life expectancy, they associate that change with a set of age–specific changes in current mortality risk valued using a set of age–specific VSMUs. To derive these VSMUs, the CIH requires data on population survival rates for each year of age, which are not available for most LMICs. They instead rely on Japanese life tables which reflect a similar change in average life expectancy at birth as found in the LMICs over the period of concern, noting that Japan provides “good historical data.” More specifically, they estimate that life expectancy at birth across all LMICs in 2000 and 2011 was 65 and 68 years, respectively. They find that Japanese life tables for 1955 and 1961 reflect a similar life expectancy at birth. CIH then uses the corresponding Japanese age–specific mortality rates to calculate changes in age–specific mortality risk for LMICs.

In its calculations, the CIH uses age groups (generally 10–year increments, although 5–year increments are used for ages 0 through 9 and a single category is used for age 80 and higher), applying values for the midpoint of each age group from the life tables. They assume that the US VSMU reflects the value for an individual aged 35, who has 45 years of remaining life expectancy, and anchor the income–adjusted values for other countries at age 35. This anchor VSMU is then adjusted for other ages in proportion to remaining life expectancy, estimated using the 1958 (midpoint of 1955 and 1961) life table for Japan.

CIH calculates the present value of the change in life expectancy by summing, over all age groups, the product of the age–specific VSMU, the age–specific risk change, and the fraction of the LMIC population in that age group in 2005 (midpoint of 2000 and 2011). Assuming the increase in life expectancy is permanent (ie, the mortality risk reduction is sustained across all future generations), CIH calculates the present value of an infinite stream of annual values using a 3 percent annual discount rate. Dividing by the increase in life expectancy yields the VLY. The CIH does not account for the age distribution shift that results from the change in mortality rates.

Prior to calculating the VLY, the CIH reduces the VSMUs for children aged 0 through 4 by 50 percent, to reflect the hypothesis that the affected populations place a lower value on these risk reductions than on those that occur at older ages. Elsewhere in its analysis, CIH tests the effects of eliminating this adjustment, of excluding children under 10, and of excluding adults older than 70.

In our calculations, we rely on data from spreadsheets provided by CIH staff, which include more detail but occasionally differ from the description in their report [2]. (It appears that the CIH intended to subtract health expenditures from the GDP per capita estimates, but does not do so consistently. However, this inconsistency has little effect on the resulting VLY.) Like the CIH, we use income as a shorthand for GDP per capita, discount future values at 3 percent per year, calculate the value of risk reductions using their estimates of the average of 2000 and 2011 GDP per capita, and calculate the VLY multiplier based on their estimate of 2000 GDP per capita. CIH references the World Bank’s 2013 World Development Indicators report as the source of the country classification used to define LMICs and the GDP estimates [25].

We explore the key assumptions listed in **Table 1**, basing our sensitivity analysis on plausible alternatives described in more detail below. Of these assumptions, we expect that the range of income elasticities will have the largest effect. The appropriate elasticity is highly uncertain, and small changes in the elasticity can alter the results by orders of magnitude.

**Table 1:** Assumptions explored in sensitivity analysis

CIH Main Case	Sensitivity Analysis
1. Assume VSMU is 1.8 percent of GDP per capita.	1. Assume VSMU is 1.7 percent of GDP per capita.
2. Assume an income elasticity of 1.0.	2(a,b). Assume an income elasticity of 1.5 or 2.0.
3(a). Convert increases in life expectancy to changes in age-specific mortality rates using Japanese life tables.	3(a). Replace Japanese life tables with Bulgarian life tables.
3(b). Adjust the VSMU for age by the ratio of the remaining life expectancy at each age and at the anchor age of 35, which is the age at which US life expectancy is 45 years.	3(b). Replace the anchor age of 35 with the age at which remaining Bulgarian life expectancy is 45 years (age 25).

CIH – Commission on Investing in Health, VSMU – value of a standardized mortality unit, GDP – gross domestic product

## SENSITIVITY ANALYSIS RESULTS

We change each assumption individually then examine their joint effect. In addition, we test the effect of re–ordering the steps, calculating the VLY for the US then adjusting for income. We do not test the effects of altering the assumption that changes in value are proportional to changes in life expectancy, although this assumption is inconsistent with much of the VSL research summarized above.

### Assumption 1: VSMU

In a recent criteria–driven literature review, the authors report a central US VSL estimate of US$ 9 million, expressed in 2013 dollars at 2013 income levels [8]. Dividing by 10 000 yields a VSMU of US$ 900; 1.7 percent of 2013 US GDP per capita in 2013 dollars. This small reduction decreases the LMIC VLY from 2.3 to 2.1 GDP per capita.

### Assumption 2: income elasticity

We apply income elasticities of 1.5 and 2.0 to illustrate the effects of larger elasticities using an equation based on the average of 2000 and 2011 US GDP per capita (US$ 44 781 and US$ 48 112, respectively) from the CIH spreadsheets [13].

VSMU_LMICs_ = VSMU_US_ × (GDP per capita_LMICs_/GDP per capita_US_)^Income Elasticity^

Because changing the elasticity has an exponential effect, the range of elasticities found in the literature significantly affects the estimates. Increasing income elasticity to 1.5 and 2.0 decreases the LMIC VLY to 0.6 and 0.2 GDP per capita, respectively, violating the expectation that individuals will value an increase in life expectancy by an amount that exceeds what they would earn over the same time period.

### Assumption 3: life tables and anchor age

We replace the Japanese life tables with life tables from a LMIC that yields a similar change in life expectancy but with a different pattern of age–specific mortality. We selected the Bulgarian life tables from 1955 and 1958 as an illustrative example because they are from the same data source as the Japanese life tables; this source includes life tables for only one other LMIC (Ukraine) [26]. We also consider the effect of changing the anchor age from 35 years to the age at which remaining life expectancy is 45 years (as assumed by the CIH for the US). For Bulgaria, this is age 25 using the midpoint (1956 or 1957) life table. Changing the life tables while keeping the anchor age at 35 reduces the GDP per capita multiplier to 1.7. Moving the anchor to age 25 leads to a further decline to 1.4.

**Figure 1:**
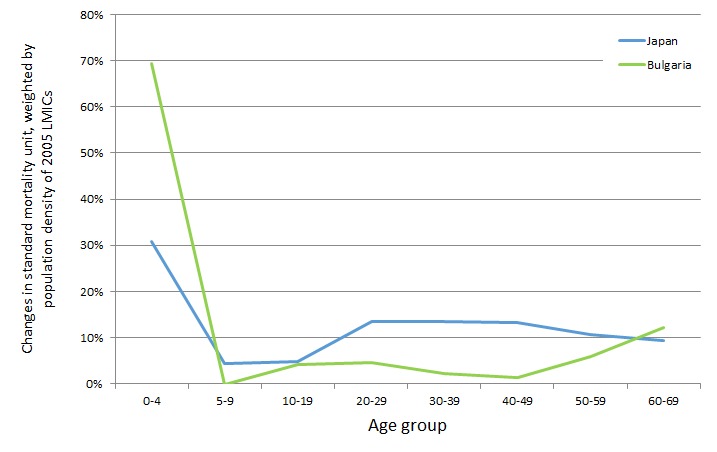
Distribution of the changes in the standard mortality unit by age group in Japan (between 1955 and 1961) and Bulgaria (between 1955 and 1958), weighted by the population density of low– and middle–income countries (LMICs) in 2005.

This analysis suggests that the VLY is sensitive to the age distribution of the survival gains. Improvements in Bulgarian survival rates between 1955 and 1958 were more concentrated in younger age groups than in Japan. We illustrate the distribution in Figure 1, which calculates the change in age–specific SMUs using each set of life tables and weights the results by the LMIC population within each age group.

### Combined effect

We combine a VSMU of 1.7 percent of GDP per capita, an income elasticity of 1.5, and Bulgarian life tables anchored at 25 years of age, and find the VLY decreases to 0.4 times GDP per capita.

Repeating each analysis without the reduction for ages 0 through 4 used in the original CIH estimates increases the estimates in each case, as expected. **Table 2** summarizes these results.

**Table 2:** Results with alternative assumptions

Sensitivity analysis	LMIC VLY as multiplier of GDP per capita	
With 50% reduction for ages 0 through 4	Without reduction for ages 0 through 4
Original CIH estimate	2.3	3.0
(1) VSMU = 1.7% of GDP per capita	2.1	2.8
(2a) Income elasticity = 1.5	0.6	0.8
(2b) Income elasticity = 2.0	0.2	0.2
(3a) Bulgarian life table, anchoring at age 35	1.7	2.9
(3b) Bulgarian life table, anchoring at age 25	1.4	2.4
Combination: VSMU = 1.7% GDP per capita, income elasticity = 1.5, Bulgarian life table anchoring at age 25	0.4	0.6

LMIC – low– and middle–income country, VLY – value of a life year, CIH – Commission on Investing in Health, VSMU – value of a standardized mortality unit, GDP – gross domestic product

### Alternative sequencing

The CIH adjusts the VSMU for income then calculates the VLY. We reverse the order, calculating VLY for the US (using US life tables from the same data source [26]) for the same period (2000 and 2011), then adjusting for income based on the average of the LMIC 2000 and 2011 values. Without reducing the values for young children, we find that the VLYs for LMICs range from 0.1 to 2.0 GDP per capita, depending on the income elasticity (**Table 3**).

**Table 3:** Results with alternative sequencing

Sensitivity analysis starting with a US VLY	LMIC VLY as multiplier of GDP per capita, without reduction for ages 0 through 4	
Without income floor	With income floor
(a) Income elasticity = 1.0	2.0	2.0
(b) Income elasticity = 1.5	0.5	1.3
(c) Income elasticity = 2.0	0.1	1.3

LMIC – low– and middle–income country, VLY – value of a life year, VSMU – value of a standardized mortality unit, GDP – gross domestic product

As in some of our earlier calculations, the resulting VLY is less than GDP per capita for LMICs when income elasticities of 1.5 and 2.0 are applied, contrary to theoretical expectations. Thus we explore the effect of using GDP per capita as a lower bound. To be consistent with the CIH approach, we set the lower bound at the average of 2000 and 2011 GDP per capita, and express the VLY as a multiplier of 2000 GDP per capita. We find the multiplier becomes 1.3 to 2.0, depending on the income elasticity. Using the average of 2000 and 2011 GDP per capita as the base would decrease the multiplier.

## DISCUSSION

The CIH methodology is fundamentally different from the traditional VSLY approach. Rather than deriving a VSLY from a VSL estimate then applying it to predicted life expectancy gains, the CIH estimates the value of age–specific changes in risks, weights by the population age distribution, then divides by the population life expectancy gain (assuming it is permanent) to calculate the average VLY.

For example, using the CIH methodology, the VLY for the change in US life expectancy from 2000 to 2011 is roughly US$ 68 000. Using the conventional approach to calculate a VSLY from a VSL yields a much larger value. The CIH assumptions yield a US VSL of US$ 7.5 million (180 times the average of 2000 and 2011 US GDP per capita). Dividing by an average remaining life expectancy of 45 years yields a VSLY of US$ 170 000, or US$ 300 000 if future years are discounted using the CIH rate of 3 percent annually. Thus the choice of approach may significantly affect the analytic conclusions, but without more empirical research it is difficult to determine which approach is most appropriate. Both have significant limitations and illustrate the enormous challenges that analysts face, given the inconsistencies and gaps in the research literature and the many ways in which they can be addressed.

The CIH approach is designed to compare the value of the historical life expectancy gains over time, across countries and regions, or across interventions. The resulting VLY estimate is an analytic *output*; it is not a value intended to be used as an *input* to value life expectancy gains in other settings. The estimates underlying the average VLY vary by country and age group, and reflect survival gains over a particular time period. Thus the 2.3 times GDP per capita value should not be directly applied in other settings.

Instead, analysts interested in applying the CIH approach would need to follow the same steps in their calculations. We find that the CIH estimate of the average VLY is very sensitive to the underlying assumptions, ranging from 0.2 to 3.0 times GDP per capita. Thus analysts should examine the inputs and assumptions and adapt them to their setting. Note, however, that multipliers less than 1.0 times GDP per capita (for the same year) seem implausible, given that we expect the VLY (as well as the VSLY) to exceed annual income. Thus GDP per capita should be used as a lower bound. Our results also suggest that analysts should explicitly address uncertainties in these values and discuss the implications for decision–making.

More specifically, as illustrated in our sensitivity analysis, the value of changes in life expectancy in LMICs is highly sensitive to the income elasticity used. However, our reviews of the literature suggest that more work is needed to determine the appropriate elasticity [13]. In the interim, analysts should test the sensitivity of their conclusions to a reasonable range of elasticities. In some cases, they are likely to find that an intervention is cost–beneficial, or is not cost–beneficial, regardless of the elasticity estimate; in other cases, the conclusions may be more uncertain. Highlighting such findings will aid decision–makers and other stakeholders in understanding the degree to which they should have confidence in the results.

The relationship of the VLY or VSLY to age or life expectancy is more complex and more difficult to resolve, raising both empirical issues (whether individuals’ willingness to pay for their own risk reductions varies by age) and normative issues (how society should value risk reductions depending on the age of those affected). As discussed earlier, the available VSL research is not conclusive, but suggests that adults in high–income countries may place higher values on risk reductions that accrue to children, that adult values for their own risk reductions over their working years may follow an inverse–U pattern, and that the values held by older adults may remain constant or decrease with age. It is unclear whether similar patterns are likely to hold in LMICs, given cultural and other differences.

Both the CIH VLY approach and the standard VSLY approach simplify this age relationship. In its main results, the CIH down–weights the values for young children, but otherwise both the VLY and the VSLY approach lead to total values that decrease with age. More work is needed to examine the empirical and normative concerns that underlie these age adjustments. In the interim, analysts should be clear about both the rationale for their approach and the extent to which it is supported by available research, and discuss the implications of related uncertainties.

Our sensitivity analysis is not intended to be a comprehensive assessment of the effects of the CIH assumptions, nor do we intend to endorse or propose any particular approach. The limited and inconsistent empirical research means that there are numerous ways in with these values can be estimated. More studies are needed to estimate key inputs such as the appropriate VSL and the relationship between VSL, age, life expectancy, and income.

## References

[1] Jamison DT, Summers LH, Alleyne G, Arrow KJ, Berkley S, Binagwaho A (2013). Global health 2035: a world converging within a generation.. Lancet.

[2] Commission on Macroeconomics and Health. Macroeconomics and Health: Investing in Health for Economic Development. Prepared for the World Health Organization. 2001.

[3] World Health Organization. The World Health Report 2002: Reducing Risks, Promoting Healthy Life. Geneva: World Health Organization. 2002.

[4] Robinson LA Hammitt JK Chang AY Resch S Understanding and improving the one and three times GDP per capita cost-effectiveness thresholds Health Policy Plan 2016Jul 24. pii: czw096. [Epub ahead of print] 10.1093/heapol/czw096 27452949

[5] Hammitt JK (2000). Valuing mortality risk: theory and practice.. Environ Sci Technol.

[6] Robinson LA, Hammitt JK (2013). Skills of the trade: valuing health risk reductions in benefit-cost analysis.. J Benefit Cost Anal.

[7] Jones-Lee M, Chilton S, Metcalf H, Nielsen JS (2015). Valuing gains in life expectancy: clarifying some ambiguities.. J Risk Uncertain.

[8] Robinson LA, Hammitt JK (2016). Valuing reductions in fatal illness risks: implications of recent research.. Health Econ.

[9] Robinson LA, Hammitt JK (2015). Research synthesis and the value per statistical life.. Risk Anal.

[10] Viscusi WK, Aldy JE (2003). The value of a statistical life: a critical review of market estimates throughout the world.. J Risk Uncertain.

[11] Robinson LA, Hammitt JK. The value of reducing air pollution risks in sub-Saharan Africa. 2009. Available: http://www.regulatory-analysis.com/robinson-hammitt-air-pollution-africa.pdf. Accessed: 1 August 2015.

[12] Lindhjem H, Navrud S, Braathen NA, Biausque V (2011). Valuing mortality risk reductions from environmental, transport, and health policies: a global meta-analysis of stated preference studies.. Risk Anal.

[13] Hammitt JK, Robinson LA (2011). The income elasticity of the value per statistical life: transferring estimates between high and low income populations.. J Benefit Cost Anal.

[14] Hammitt JK (2007). Valuing changes in mortality risk: lives saved versus life years saved.. Rev Environ Econ Policy.

[15] Hammitt JK (2013). Admissible utility functions for health, longevity, and wealth: integrating monetary and life-year measures.. J Risk Uncertain.

[16] Aldy JE, Viscusi WK (2007). Age differences in the value of statistical life: revealed preference evidence.. Rev Environ Econ Policy.

[17] Aldy JE, Viscusi WK (2008). Adjusting the value of a statistical life for age and cohort effects.. Rev Econ Stat.

[18] Cameron TA, DeShazo JR, Stiffler P (2010). Demand for health risk reductions: A cross-national comparison between the U.S. and Canada.. J Risk Uncertain.

[19] Evans MR, Smith VK (2006). Do we really understand the age-VSL relationship?. Resour Energy Econ.

[20] Krupnick A (2007). Mortality-risk valuation and age: stated preference evidence.. Rev Environ Econ Policy.

[21] Hammitt JK, Haninger K (2010). Valuing fatal risks to children and adults: effects of disease, latency, and risk aversion.. J Risk Uncertain.

[22] Alberini A, Loomes G, Scasny M, Bateman I. Valuation of environment-related health risks for children. Paris: OECD Publishing; 2010.

[23] Blomquist GC, Dickie M, O’Conor RM (2011). Willingness to pay for improving fatality risks and asthma symptoms: values for children and adults of all ages.. Resour Energy Econ.

[24] Cameron TA (2010). Euthanizing the value of a statistical life.. Rev Environ Econ Policy.

[25] World Bank. World Development Indicators, 2013. Washington, DC: World Bank.

[26] Human Mortality DatabaseAvailable: http://www.mortality.org. Accessed: 1 August 2015.

